# Characterization of a Microalgal UV Mutant for CO_2_ Biofixation and Biomass Production

**DOI:** 10.1155/2018/4375170

**Published:** 2018-12-23

**Authors:** Feng Qi, Daoji Wu, Ruimin Mu, Shuo Zhang, Xinyi Xu

**Affiliations:** ^1^School of Municipal and Environmental Engineering, Shandong Jianzhu University, Jinan 250101, China; ^2^Shandong Provincial Engineering Centre on Environmental Science and Technology, Jinan 250061, China; ^3^College of Environmental Science and Engineering, Ocean University of China, Qingdao 266100, China; ^4^Shandong Co-Innovation Center of Green Building, Jinan 250101, China; ^5^Shandong Geomineral Engineering Group Co. Ltd., Jinan 250200, China; ^6^School of Environmental Science and Engineering, Shandong University, Jinan 250100, China

## Abstract

The mutagenesis is an emerging strategy for screening microalgal candidates for CO_2_ biofixation and biomass production. In this study, by 96-well microplates-UV mutagenesis, a mutant stemmed from* Scenedesmus obliquus* was screened and named as SDEC-1M. To characterize SDEC-1M, it was cultivated under air and high level CO_2_ (15% v/v), and its parental strain (PS) was considered as control. Growth characterizations showed that SDEC-1M grew best in high level CO_2_. It indicated that the mutant had high CO_2_ tolerance (HCT) and growth potential under high level CO_2_. Richer total carbohydrate content (37.26%) and lipid content (24.80%) demonstrated that, compared to its parental strain, SDEC-1M was apt to synthesize energy storage materials, especially under high CO_2_ level. Meanwhile, the highest light conversion efficiency (approximately 18 %) was also obtained. Thus, the highest overall biomass productivities were achieved in SDEC-1M under high level CO_2_, largely attributed to that the highest productivities of total lipid, total carbohydrate, and crude protein were also achieved in the meantime. By modified UV, therefore, mutagenized SDEC-1M was the better candidate for CO_2_ biofixation and biofuel production than its parental strain.

## 1. Introduction

Energy shortage and climatic change have been greatly focused [1–3]. To solve the problems, the techniques of CO_2_ capture and sequestration and clean energy alternative to fossil fuel have been widely applied, among which microalgae is one of the most interesting strategies due to being sustainable, environment-friendly, and noncompetitive with other edible feedstocks [1, 4–10].

Microalgae cultivation via autotrophic system with high level CO_2_ is considered as a more viable approach for commercialization due to lower cultivation costs cut by free sunlight and carbon source, high contents of lipid or carbohydrate, acquiring high cell density and sequestrating CO_2_ [1, 7, 8, 11]. However, the growth of microalgae can also be inhibited by high level CO_2_, due to acidified medium [12]. Thus, an ideal candidate should have tolerance for high level CO_2_. In previous reports, in addition to the isolation of microalgae from the region affected by atmospheric pollution source [1] and acclimation by bubbled gas with CO_2_ gradually increasing [13], screening microalgae with high CO_2_ tolerance (HCT) by genetic strategy coupled to high-throughput screening were emerging [3, 4, 13–17]. In consideration of the economic and technological feasibility of genetic manipulations [3, 13, 15], random mutagenesis was more employed to provide microalgae candidates, rather than targeted genetic manipulation [17]. For instance, some mutants with HCT were obtained by chemical [4], nuclear irradiation [13], plasmas [18], and UV mutagenesis [14, 15]. Following mutagenesis, general, surviving mutants should be isolated [4, 15], which means enormous work and risks from invasion of infectious bacteria and algae. According to novel 96-well microplates-UV mutagenesis, each cell was isolated and then mutagenized in a well of 96-well microplates [14]. Since each surviving colony in the closed well was pure strain, luckily, invasion and reisolation were avoided. The novel method can shorten operating time, simplify operation process, and maintain purity of isolated strains [14].

Mutants can access some characteristics which their parental strains do not have, for instance, high biomass productivities [17], high abilities to capture CO_2_ [4], high light conversion efficiencies (*LCE*) [14, 17, 19], high CO_2_ requiring (HCR) [14, 20], and high contents of lipid or carbohydrate [14–16]. These genetic characteristics affecting their ability to biofix CO_2_ and product biomass are key to commercialize microalgae [16]. In view of randomicity of mutagenesis, the above-mentioned characteristics might synchronize. Therefore, it is necessary that the mutants are characterized to test their commercialization potential of the CO_2_ biofixation and biomass production.

In this study, we aimed to have microalgae strains for CO_2_ biofixation and biomass production;* Scenedesmus obliquus* was mutagenized by 96-well microplates-UV mutagenesis. One competitive potential mutant was screened out. To characterize its growth, biochemical components, and* LCE*, moreover, the mutant and its parental strain were cultivated under air and high level CO_2_ (15% v/v). After continuous subculture for five generations under 15% CO_2_, its genetic stability was tested. According to the experimental results, finally, the mutant's suitability for CO_2_ fixation and application of its biomass were evaluated.

## 2. Material and Methods

### 2.1. Microalgae Strains and Culture Medium

In this study, as a parental strain (PS), one freshwater microalgae* Scenedesmus obliquus* observed dominant in freshwater water systems [27] was obtained from FACHB-Collection of Institute of Hydrobiology, Chinese Academy of Sciences. By 96-well microplates-UV mutagenesis [14], several* S. obliquus* mutants survived in wells with low pH (4.5) BG11 culture medium, and the survival ratio was approximately 3%. The growth status and genetic stabilities of survivors cultivated under 15% (v/v) CO_2_ were compared and the best-growing and good genetically stable mutant was selected and named as SDEC-1M and employed in this study. The modified SE media (Brostol's solutions) with 1000 mg L^−1^ of NaNO_3_ nitrate concentrations [28] were used, which can supply more nitrogen source during the cultivation period. In addition to NaNO_3_, there are 75 mg of K_2_HPO_4_·3H_2_O, 75 mg of MgSO_4_·7H_2_O, 25 mg of CaCl_2_·2H_2_O, 175 mg of KH_2_PO_4,_ 25 mg of NaCl, 5 mg of FeCl_3_·6H_2_O, 1mL of A_5_ solution, 1 mL of Fe-EDTA, and 40 mL of soil extract in 958 mL of deionized water. 1L A_5_ solution contained 2.86 g of H_3_BO_3_, 1.81 g of MnC1_2_·4H_2_O, 0.22 g of ZnSO_4_·4H_2_O, 79 mg of CuSO_4_·5H_2_O, and 39 mg of (NH)_6_Mo_7_O_24_·4H_2_O. 1L Fe-EDTA contained 10g of Na_2_EDTA, 0.81 g of FeCl_3_·6H_2_O, and 500 mL of 0.1 M HCl. The soil extract was the filtered supernatant from boiled soil solution.

### 2.2. Culture Conditions

The parental strain cells or mutant cells were cultured in photobioreactors (inside diameter (ID), 120 mm; working height (H_w_), 221 mm; working volume (V_w_, 2.5L)) [22] containing fresh medium. Then, they were kept in a phytotron at 25 ± 1°C. A continuous illumination was provided by a row of fluorescent lamps which were horizontally fixed on the wall at one side of the bottles. The illumination intensities on the photobioreactor surface toward and back to the light source were 47.25 and 2.7 *μ*mol m^−2^s^−1^, respectively, read by a photometer. The initial OD_686_ (optical density at 686 nm) was 0.3 (approximately 73 mg L^−1^ of biomass concentration). During cultivation, deionized water was added to keep working volume. Aeration was carried out by air or high level CO_2_. Simulating flue gas with high level CO_2_ (up to 15%, v/v) [10], 15% (v/v) CO_2_ mixed by air and pure CO_2_ that were prearranged in industrial cylinders were employed. Using gas flow meters (Sevenstar, Beijing, China), the flow rates were adjusted to 0.2 vvm (volume gas per volume culture per min) of air and 0.04 vvm of mixture, respectively.

### 2.3. Measurement Methods

Between 680 and 690 nm, Chang and Yang [29] and Akkerman [30] found absorption peaks of microalgal broth and a linear correlation between the biomass concentrations and the optical densities. Thus, the indirect estimation of biomass concentration by optical density (OD) between 680 and 690 nm has been widely used in the microalgal research [2, 14, 28]. The absorption peaks of PS and SDEC-1M were located at 686nm by spectral scanning with a UV-2450 spectrophotometer (SHIMADZU, Japan). OD_686_ and the biomass concentration of PS and SDEC-1M broth diluted in different proportions were determined by a UV-2450 spectrophotometer (SHIMADZU, Japan) and weighing the dry mass, respectively. Then, (1) relating OD_686_ to biomass concentration was established by the linear regression.

(1) $$\begin{array}{c} X={OD}_{686}\times 254.1-3.182\;R^{2}=0.9927 \end{array}$$

Sample was taken once every day. After proper dilution, the biomass concentrations (*X*, mg L^−1^) of sample were indirectly calculated via (1). pH of the sample was determined using PHS-3C pH meter (Leici, Shanghai, China).

At the end of cultivation (day 7), the microalgae were harvested and the contents of their primary biochemical components were measured. Microalgal pellets were formed by centrifuging microalgae culture at 4000 rpm at -3°C for 10 min and were washed twice with 0.5 M of ammonium formate to desalinate. Then, the microalgal pellets were dried and ground into powder. Higher heating values (HHV) of microalgal powder were determined by an isotherm oxygen bomb calorimeter [31]. Lipids of the microalgae were extracted by solvent, and the total lipid contents were estimated gravimetrically using a modified method [27]. The total carbohydrate contents of the microalgae were measured by the phenol-sulfuric acid method [32]. The crude protein content in the biomass was calculated via (2).

(2) $$\begin{array}{c} \text{Protein content}=\text{Nitrogen content}\times 6.25 \end{array}$$

where nitrogen content was measured according to the Kjeldahl method [33], and the factor 6.25 is the correlation between protein content and nitrogen content reported by previous studies [34, 35].

### 2.4. Calculations on Important Properties

The maximum biomass concentration (mg L^−1^) was designated as* X*_*max*_.

The overall biomass productivity (*P*_*overall*_, mg L^−1^d^−1^) was calculated via (3).

(3) $$\begin{array}{c} P_{overall}=\frac{\left(X_{7\text{-}}X_{0}\right)}{7} \end{array}$$

where* X*_*7*_ is the biomass concentration on day 7;* X*_*0*_ is the initial biomass concentration in mg L^−1^;* 7 *is the cultured time (d).

Specific growth rate (*μ*_*t*_, d^−1^) in a day was calculated via (4). The maximum specific growth rate was designated as* μ*_*max*_ (d^−1^).

(4) $$\begin{array}{c} \mu_{t}=\frac{\left(\ln X_{t}-\ln X_{t\text{-}1}\right)}{1} \end{array}$$

where* X*_*t*_ and *X*_*t*-1_ were the biomass concentration (mg L^−1^) on day t and day t-1, respectively; 1 was the time (d) gone through from day t-1 to day t.

The light conversion efficiency (*LCE*, %) based photosynthetic active radiation was estimated via (5).

(5) LCE=HHV×Poverall×Vw×100I++I-×k×PAR×A×t=2.08×10-2×HHV×Poverall

where the units of* HHV*,* P*_*overall*_ and* V*_*w*_ are J mg^−1^, mg L^−1^d^−1^ and L;* I*^*+*^ and *I*^−^ are illumination intensities (mol m^−2^s^−1^) on the photobioreactor surface toward and back to the light source, respectively; the constant* k* that converts illumination intensity to light energy density (W m^−2^) is 218800 J mol^−1^ photons [36]; the coefficient of photosynthetic active radiation (*PAR*) is 48% [37];* A* is the irradiated area (m^2^);* t* is 86400 seconds in a day.

Total lipid productivity (*P*_*L*_, mg L^−1^d^−1^), total carbohydrate productivity (*P*_*C*_, mg L^−1^d^−1^), and crude protein productivity (*P*_*P*_, mg L^−1^d^−1^) used to test potential of lipid, carbohydrate, and protein productions were calculated via (6), (7), and (8).

(6) PL=Poverall×total lipid content

(7) PC=Poverall×total carbohydrate content

(8) PP=Poverall×crude protein content

### 2.5. Statistical Analysis

The differences between parameters of PS and SDEC-1M determined under air and 15% CO_2_ were assessed using one-way analysis of variance (ANOVA). A difference was considered statistically significant when* p* < 0.05. Duncan's test was performed to detect the statistical significance of differences (p > 0.05).

## 3. Results and Discussion

### 3.1. Growth Characterizations

#### 3.1.1. Growth Characterization under Air

**Figure 1:**
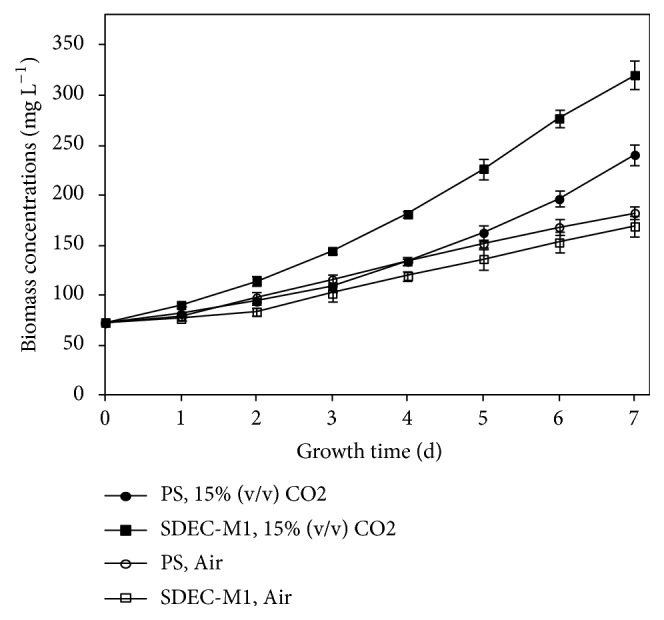
Growth curves of* S. obliquus* parental strain (PS) and mutant (SDEC-1M) under air and 15% (v/v) CO_2_ for 7 days. Each data indicates the mean ± SD, which was measured from three independent cultures.

As shown in Figure 1, PS grew slightly better than SDEC-1M under air. More details were seen in Table 1; no significant differences on* X*_*max*_,* μ*_*max*_ and* P*_*overall*_ were observed between PS and SDEC-1M under air. Thus, there were no significant changes in growth characteristics of SDEC-1M under air by mutagenesis. Due to randomicity of mutagenesis, obviously, SDEC-1M did not attain the high CO_2_ requiring (HCR) characteristic closely related to defectiveness in CO_2_ concentrating mechanisms (CCMs) [20]. Although it was confirmed that a UV mutant,* Chlorella vulgaris* SDEC-3M obtained by 96-well microplates-UV mutagenesis, was a HCR mutant [14], this method is not an efficient method for screening HCR algae strains.

#### 3.1.2. Growth Characterization under High Level CO_*2*_

Some species in* Scenedesmus *screened for CO_2_ fixation were reported [1, 24, 25, 38]. These species generally have two characteristics: survival in low pH medium caused by high level CO_2_ (high CO_2_ tolerance) and high CO_2_ fixation efficiency that exhibits good growing ability [7, 12, 13]. As shown in Figure 1, PS and SDEC-1M both exhibited higher growth rates under 15% (v/v) CO_2_, and the latter adapted to high level CO_2_ faster and attained the linear growth phase earlier. More details were seen in Table 1; thus, although there was no significant difference in* μ*_*max*_ between SDEC-1M and PS under 15% CO_2_, SDEC-1M maintained a longer logarithmic phase (from day 1 to day 6), and its* X*_*max*_ and* P*_*overall*_ were significantly 33.10% and 47.09% higher than those of PS, respectively. These results demonstrate that longer logarithmic phase can increase the biomass productivity.

#### 3.1.3. Growth Distinction under Air and High Level CO_*2*_

As shown in Figure 1, PS or SDCE-1M grew better under 15% CO_2_ than under air. More details were seen in Table 1; there were no significant differences on their* μ*_*max*_ between under air and under 15% CO_2_, while* μ*_*max*_ occurred at different times. Comparing their performances under air, the* X*_*max*_ and* P*_*overall*_ of PS and SDEC-1M were both significantly higher. It implies that their growth potentials are similar, but the performances under air are poorer. Insufficient carbon source should be the main reason to decrease the metabolism of microalgae under air [7].

### 3.2. Contents and Productivities of Biochemical Components

**Figure 2:**
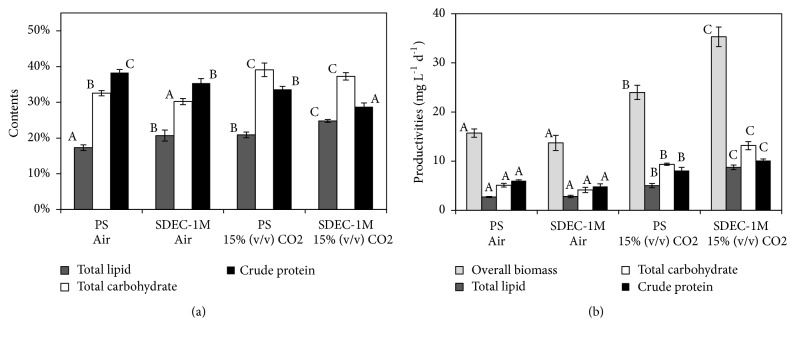
Contents of total lipid, total carbohydrate, and crude protein in cell (a) and productivities (b, mg L^−1^d^−1^) of overall biomass, total lipid, total carbohydrate, and crude protein in* S. obliquus* parental strain (PS) and mutant (SDEC-1M) under air and 15% (v/v) CO_2_ for 7 days. Each data indicates the mean ± SD, which was measured from three independent cultures. Data of the same component followed by different letters are significantly different by Duncan's test (*p* < 0.05).

Due to their plenty compounds as lipids, carbohydrates, and protein, microalgae have enormous potential for the sustainable production of food, fuels, and other biochemicals [17]. Compounds contents and productivities are essential parameters of evaluation on applied purposes of microalgae biomass [23]. As shown in Figure 2(a), whether under air or 15 % CO_2_, the protein contents in PS were significantly higher than their lipid and carbohydrate contents, which agreed with recent reports, representing 32-58% of protein contents in* Scenedesmus* sp. [5, 23, 26, 39, 40]. However, in mutant SDEC-1M, the contents of lipid and carbohydrate were significantly higher than in PS, while the protein contents were just the opposite. Thus, carbohydrates became the chief biochemical component in SDEC-1M. Meanwhile, the lipid contents in PS and SDEC-1M under 15 % CO_2_ were both significantly higher than those under air. It agrees with the previous findings that high CO_2_ stress enhanced lipid production [7, 41]. These results imply that compared to its parental strain, SDEC-1M was apt to synthesize energy storage materials, especially under high CO_2_ level.

Largely attributed to significant higher overall biomass productivities, the highest productivities of total lipid (8.76 ± 0.47 mg L^−1^d^−1^), total carbohydrate (13.16 ± 0.82 mg L^−1^d^−1^), and crude protein (10.14 ± 0.30 mg L^−1^d^−1^) all occurred in SDEC-1M under 15 % CO_2_, even though crude protein content was lower. Considering that two strains were harvested in logarithmic phase or linear growth phase, the results imply that biomass productivity plays a leading role in the primary biochemical components production until stationary phase, which agreed with previous reports [2, 6, 42].

### 3.3. Genetic Stabilities under High Level CO_*2*_

**Figure 3:**
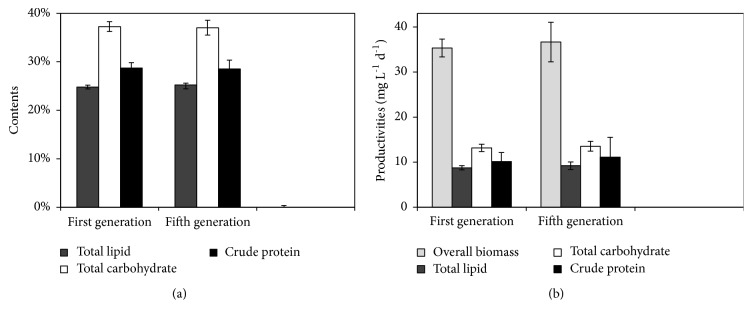
Contents of total lipid, total carbohydrate, and crude protein in cell (a) and productivities (b, mg L^−1^d^−1^) of overall biomass, total lipid, total carbohydrate, and crude protein in SDEC-1M of the first generation and the fifth generation under 15% (v/v) CO_2_ for 7 days. Each data indicates the mean ± SD, which was measured from three independent cultures.

In view of the instability of mutagenized strains, especially in continuous subculture, their genetic stability should be tested [18]. Thus, to confirm its stability of biomass product, SDEC-1M, was continuously subcultured under 15% CO_2_ for five generations. The characterized results in SDEC-1M of the first generation and the fifth generation were shown in Figure 3.

As shown in Figure 3, no significant changes were observed on parameters, such as contents of total lipid, total carbohydrate, and crude protein and productivities of overall biomass, total lipid, total carbohydrate, and crude protein in SDEC-1M between the first generation and the fifth generation. The results suggest SDEC-1M is genetically stable from the aspect of biomass production.

### 3.4. Light Conversion Efficiency (LEC)

As shown in Table 2, there was no significant difference in* HHV* between PS and SDEC-1M under air, and similar results under 15% CO_2_. Furthermore,* HHV* of SDEC-1M was significantly higher than that of PS whether under 15% CO_2_ or under air. The results were similar to their growth characterizations. Thus, the highest overall biomass productivity (35.32 ± 1.99 mg L^−1^d^−1^) (Figure 2) or the highest* HHV* (24.43 ± 0.59 KJ g^−1^) were both obtained by SDEC-1M under 15% CO_2_. As a result, the highest calculated value of* LCE *(17.93 ± 0.6 %) occurred in SDEC-1M under 15% CO_2_.

The higher* LCE* means more light energy transferred into biomass under same light conditions. Thus, the high overall biomass productivity was obtained in SDEC-1M under 15% CO_2_ (Section 3.2). Only photosynthetic active radiation (PAR) is usable radiation fraction (400–700 nm) wavelengths between 400 and 700 nm [43], accounting for 42.3%-45.8% of the total energy from the solar spectrum [44, 45]. Based on PAR,* LCEs* of most microalgae varied between 4-9% [45], just in which* LCEs* of PS and SDEC-1M were under air. A few recorded higher* LCEs *of 21.6% [19] and 14.52% [11] were comparable with those under 15% CO_2_ in this study. This result is close to the theoretical upper limit of conversion efficiencies from solar energy to chemical energy (26.7%-29.8%) previously estimated by Brennan and Owende [45] and Robertson et al. [37]. Interestingly, the higher* LCEs* all were obtained in the cases with additional CO_2_ aeration. It shows that CO_2_ level is the crucial factor for enhancing biomass production of algal strains with HCT characteristic.

### 3.5. Characteristics Comparison with Other Algae Strains

The main characteristics represented the growth potential and conversion efficiency of SDEC-1M and other algae strains were shown in Table 3. By comparison, it is found that the contents of lipid, carbohydrate, and protein of SDEC-1M are all moderate, representing the normal level of primary biochemical components in algae, especially* Scenedesmus* sp. Optimization and stress aiming at production enhancement of specific component, such as lipid [2, 22], carbohydrate [23], and protein [1, 26] were not implemented, and the cultured time is not long enough to attain the stable phase in this study. It implies that SDEC-1M has yet great potential to enhance production of lipid, carbohydrate, and protein.

## 4. Conclusions

*S. obliquus* SDEC-1M with genetic stability was obtained after UV mutagenesis and was cultivated under air and 15% CO_2_, with its parental strain used as control. SDEC-1M got the best growth performance and the highest* LCE* (17.93 %) under 15% CO_2_, which confirms its high CO_2_ tolerance and high CO_2_ fixation efficiency. Meanwhile, the highest total carbohydrate and lipid contents (37.26% and 24.80 %, respectively) and productivity (13.16 and 8.76 mg L^−1^d^−1^, respectively) were obtained. These results confirmed SDEC-1M's ability to convert more efficiently light into energy storage materials. Compared to its parental strain, therefore, SDEC-1M is a more suitable candidate for CO_2_ fixation and biomass production, especially biofuel production, which mitigates the global warming and energy shortage.

## Figures and Tables

**Table 1:** Maximum biomass concentrations (*X*_*max*_), maximum specific growth rates (*μ*_*max*_), and maximum biomass productivities for *S. obliquus* parental strain (PS) and mutant (SDEC-1M) under air and 15% (v/v) CO_2_ for 7 days. Each data indicates the mean ± SD, which was measured from three independent cultures. Each value in parentheses indicates the time (d) in which the maximum value of the parameter occurs. Data in the same parameter followed by different letters are significantly different by Duncan's test (*p* < 0.05).

Strains	*X*_*max*_ (mg L^−1^)		*μ*_*max*_(d^−1^)		*P*_*overall*_(mg L^−1^d^−1^)	
	Air	15% (v/v) CO_2_	Air	15% (v/v) CO_2_	Air	15% (v/v) CO_2_
PS	182.52 ± 5.85 (7)^a^	240.62 ± 10.2 (7)^b^	0.21 ± 0.05 (2)^a^	0.21 ± 0.02 (4)^a^	15.96 ± 1.29 (6)^a^	24.01 ± 3.2 (7)^b^
SDEC-1M	169.13 ± 10.74 (7)^a^	320.28 ± 13.9 (7)^c^	0.2 ± 0.02 (3)^a^	0.23 ± 0.03 (2)^a^	13.73 ± 1.89 (7)^a^	35.32 ± 1.81 (7)^c^

**Table 2:** Higher heating values (*HHV*) and light conversion efficiencies (*LCE*) for *S. obliquus* parental strain (PS) and mutant (SDEC-1M) under air and 15% (v/v) CO_2_ for 7 days. Each data indicates the mean ± SD, which was measured from three independent cultures. Data in the same parameter followed by different letters are significantly different by Duncan's test (*p* < 0.05).

Strains	*HHV*(kJ g^−1^)		*LEC*(%)	
	Air	15% (v/v) CO_2_	Air	15% (v/v) CO_2_
PS	22.03 ± 0.47^a^	23.1 ± 0.26^bc^	7.2 ± 0.29^a^	11.54 ± 0.74^b^
SDEC-1M	22.53 ± 0.35^ab^	24.43 ± 0.59^c^	6.43 ± 0.62^a^	17.93 ± 0.6^c^

**Table 3:** Comparison between characterizations of SDEC-1M and other algae strains.

Strain	Lipid content (%)	Carbohydrate content (%)	Protein content (%)	*HHV* (kJ g^−1^)	*LEC* (%)	Reference
SDEC-1M	24.80	37.26	28.74	24.43	17.93	This study
*Chlorella protothecoides*	-	-	-	21.1	-	[21]
*Chlorella* sp.	33.6	-	-	-	-	[22]
*Chlorella vulgaris*	32.7	-	-	-	-	[2]
*Chlorella vulgaris *SDEC-3M	19.15	42.48	-	22.1	14.52	[14]
*Cladophora fracta*	-	-	-	25.1	-	[21]
*Phaeodactylum tricornutum *UTEX 640	-	-* *-	-	-	21.6	[19]
*Scenedesmus bajacalifornicus* BBKLP-07	25.81	26.19	32.89	-	-	[1]^a^
*Scenedesmus dimorphus*	12	53.7	17.4	-	-	[23]
*Scenedesmus obliquus*	19.80	25.39	31.26	-	-	[2]
*Scenedesmus obliquus*	27.5	-	-	-	-	[24]
*Scenedesmus obliquus*	-	-	-	22.9	-	[25]
*Scenedesmus* sp.	21	38	23	-	-	[5]
*Scenedesmus* sp.	25.4	-	49.97	-	-	[26]^a^

a: the optimal value under different conditions.

b: unit is 10^6^ cell/mL.

## Data Availability

The data used to support the findings of this study are available from the corresponding author upon request.

## Abbreviations List

UV: Ultraviolet
PS: Parental strain
HCT: High CO_2_ tolerance
LCE: Light conversion efficiencies
HCR: High CO_2_ requiring
OD: Optical density
HHV: Higher heating values
PAR: Photosynthetic active radiation
ANOVA: Analysis of variance.
